# Design of Highly Specific Antimicrobial Peptides Targeting the BamA Protein of *Candidatus* Liberibacter Asiaticus

**DOI:** 10.1021/acsomega.5c11153

**Published:** 2026-02-02

**Authors:** Samavath Mallawarachchi, Sonia Irigoyen, Kranthi Mandadi, James Borneman, Sandun Fernando

**Affiliations:** † Department of Biological and Agricultural Engineering, 14736 Texas A&M University, College Station, Texas 77843, United States; ‡ Texas A&M AgriLife Research & Extension Center, 57804 Texas A&M University System, 2415 E. Highway 83, Weslaco, Texas 78596, United States; § Department of Plant Pathology and Microbiology, Texas A&M University, College Station, Texas 77843, United States; ∥ Institute for Advancing Health Through Agriculture, Texas A&M AgriLife, College Station, Texas 77843, United States; ⊥ Department of Microbiology & Plant Pathology, 8790 University of California Riverside, Riverside, California 92507, United States

## Abstract

*Candidatus* Liberibacter asiaticus (CLas) is a putative causative agent of Huanglongbing (citrus greening). The unculturable nature of CLas poses a significant challenge in discovering drugs against citrus greening. This study presents a novel in silico technique to design peptides with a high affinity toward the β-barrel assembly machinery A (BamA) protein, a critical outer membrane component of CLas vital for bacterial functionality. The technique used in this study is based on identifying the strongest binding amino acids at different sites in BamA and linking them using peptide linkers. Initially, amino acid probes that can emulate amino acid activity in peptide form were docked using Schrodinger Glide on the target domain of BamA. Docking results of amino acid probes showed three closely located clusters on BamA. Peptides were designed by selecting the strongest binding probes in each cluster and linking them using short peptide linkers. Initially, 15 peptides were designed, and based on molecular docking, molecular dynamics simulations, and BioLayer Interferometry, three peptides with a high affinity toward BamA were identified. Two of the peptides effectively inhibited *Rhizobium grahamii*, a CLas surrogate, in in vitro assays, suggesting potential antimicrobial activity against CLas.

## Graphical abstract

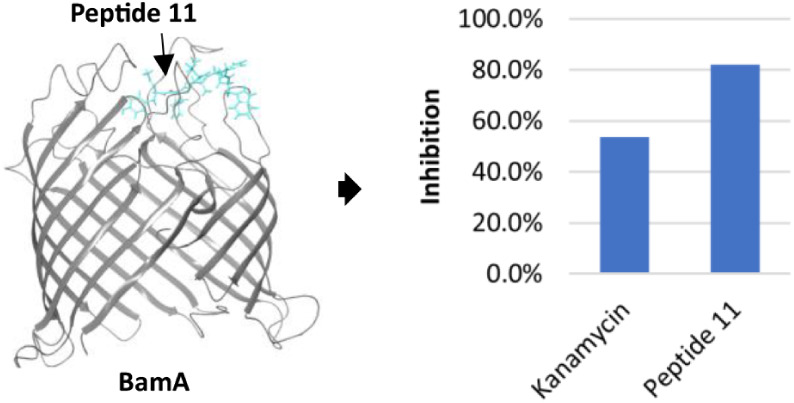

## Introduction

Citrus greening or Huanglongbing (HLB) is a severe disease that has greatly affected citrus plantations worldwide.^1^ The impact of this disease has been most severe in Florida, where a 75% reduction in citrus production has resulted in estimated losses of over 1 billion US dollars per year and the loss of more than 5000 jobs per year.^2^ This disease has been attributed to three Gram-negative bacterial strains of the *Candidatus* Liberibacter genus: *Candidatus* Liberibacter asiaticus (CLas), C*andidatus* liberibacter africanus (CLaf), and *Candidatus* liberibacter americanus (CLam). CLas is the most prevalent and aggressive strain, and it is widespread in the United States, Asia, and South America, and has also spread to some regions in Africa.^3^ Transmission of these bacteria occurs through psyllid vectors, specifically *Diaphorina citri* (Asian citrus psyllid) for CLas and CLam, and *Trioza erytreae* (African psyllid) for CLaf.^3–6^ Secretion of virulent effector proteins by the causative bacteria leads to the death and malfunctioning of phloem tissues.^7,8^

Currently, HLB is considered incurable, and all commercially available citrus species are susceptible to HLB.^9,10^ Removing infected trees and controlling psyllids are the most widely used HLB management strategies and have shown limited effectiveness.^11–13^ While trunk injection and foliar application of conventional antibiotics, such as streptomycin, oxytetracycline, and penicillin, have demonstrated significant efficacy against HLB, this is not considered a sustainable solution due to the high risk of antibiotic resistance.^14–20^ Several studies have been conducted on enhancing the resistance of citrus trees to HLB via cross-breeding or genetic modification.^10,21–28^ Nevertheless, these studies are still in the developmental stage, emphasizing the critical need to discover an effective treatment for HLB.

CLas is a phloem-limited bacterium and cannot be cultured under normal conditions, which presents a significant challenge in developing drugs against HLB.^29–33^ Due to the labor-intensive and time-consuming nature of screening drugs via in-planta trials, alternative drug screening techniques, such as in silico simulations or the use of culturable surrogates, are widely used.^7,34,35^

This study discusses a novel in silico technique to design peptides with a high affinity toward the BamA protein of CLas bacteria. The BamA protein is an essential component of the β-barrel assembly mechanism (BAM), which facilitates the proper folding of outer membrane proteins (OMPs) and their insertion into the outer membrane.^36–41^ These OMPs play a crucial role in multiple cellular functions, including nutrient absorption, toxin excretion, and adhesion in Gram-negative bacteria.^37–39^ Thus, the BamA protein is essential for bacterial functioning, making it an ideal target for drug screening purposes.

Antimicrobial peptides (AMPs) have recently attracted attention as promising approaches against CLas. AMPs are short sequences of amino acids that have the potential to inhibit bacterial action. AMPs act as natural defense molecules in a diverse range of organisms, including plants, animals, and fungi.^42^ Some AMPs are reported to enhance the innate immune response in the hosts, and most AMPs can inhibit the growth or action of pathogenic microorganisms through mechanisms such as disruption of cell membranes and manipulation of ion channels.^42–46^ Advantages of AMPs compared to conventional antibiotics include a broader range of action, the ability to overcome resistance, and better environmental compatibility.^43,46,47^ It is reported that AMPs are less prone to resistance compared to small-molecule antibiotics, and the resistance to AMPs can be overcome by subtle structural modifications of the AMPs.^48,49^ Several studies have reported the successful use of AMPs or other small molecules to inhibit CLas within citrus leaves and enhance the immune response in citrus trees,^31,50–52^ while another study has reported several natural and synthetic AMPs that have been effective against surrogates closely related to CLas.^34^ In addition, increased expression of AMPs, including defensins and thionins, has been observed to enhance the resistance of citrus against HLB.^21,23,25^

Researchers have used multiple approaches to design AMPs targeting CLas, including identifying common AMP sequences in HLB-tolerant varieties, screening of existing peptides that are reported to have shown antimicrobial activity, and structural modifications of known AMPs.^7,31,33,34,50^ This study presents a novel in silico technique to design potential AMPs with a high affinity toward BamA. This technique is based on identifying the amino acids that would bind most strongly to the desired domain in BamA and developing peptide structures consisting of those amino acids.

## Results

### Docking of Modified Amino Acids

To identify the amino acids with the highest affinity to BamA, amino acid probes, which were modified to emulate the behavior of amino acids with peptide bonds, were docked to BamA. The binding of modified amino acids to BamA was analyzed based on docking scores, Glide binding energies, binding sites, and interactions. The docking scores, Glide binding energies, and main residues in BamA that interact with each amino acid are provided in Table 1, and the binding sites of the amino acids on BamA are visualized in Figure 1.

**Table 1:** Docking Scores, Glide Energies, and Interactions with BamA of Each Amino Acid Probe

Amino acid	Docking score (kcal/mol)	Glide energy (kcal/mol)	Binding cluster	Residues in BamA forming H bonds
VAL	–6.337	–24.719	2	ALA720, ASN721, HIS722, ASP734
THR	–5.818	–27.418	2	ALA720, ASN721, HIS722, ASP734
TRP	–5.199	–27.487	3	
HIS	–5.165	–24.138	3	LEU652, ILE682
TYR	–5.046	–20.188	1	LYS558, VAL723
ASN	–4.616	–22.410	3	LEU652, PHE653
PRO	–4.487	–18.163	3	ASP515, LYS670
ILE	–4.057	–18.527	3	ASP515
GLN	–4.033	–22.976	3	LEU652, PHE653
SER	–3.968	–20.389	3	ASP515, LEU652
CYS	–3.962	–20.168	3	LEU652, PHE653
ALA	–3.901	–21.662	2	ALA720, ASN721, HIS722, ASP734
LEU	–3.769	–18.661	3	ASP515
PHE	–3.675	–16.647	1	
ASP	–3.667	–15.220	2	HIS722, ASN733
MET	–3.517	–20.914	3	ASP515
GLU	–3.335	–18.709	2	ALA720, ASN721, ASN733
ARG	–3.087	–22.395	3	ASP515, LEU652, TYR669
LYS	–2.948	–22.112	3	ASP515, LEU652
GLY	–2.881	–19.414	2	ALA720, ASN721, HIS722, ASP734

**Figure 1:**
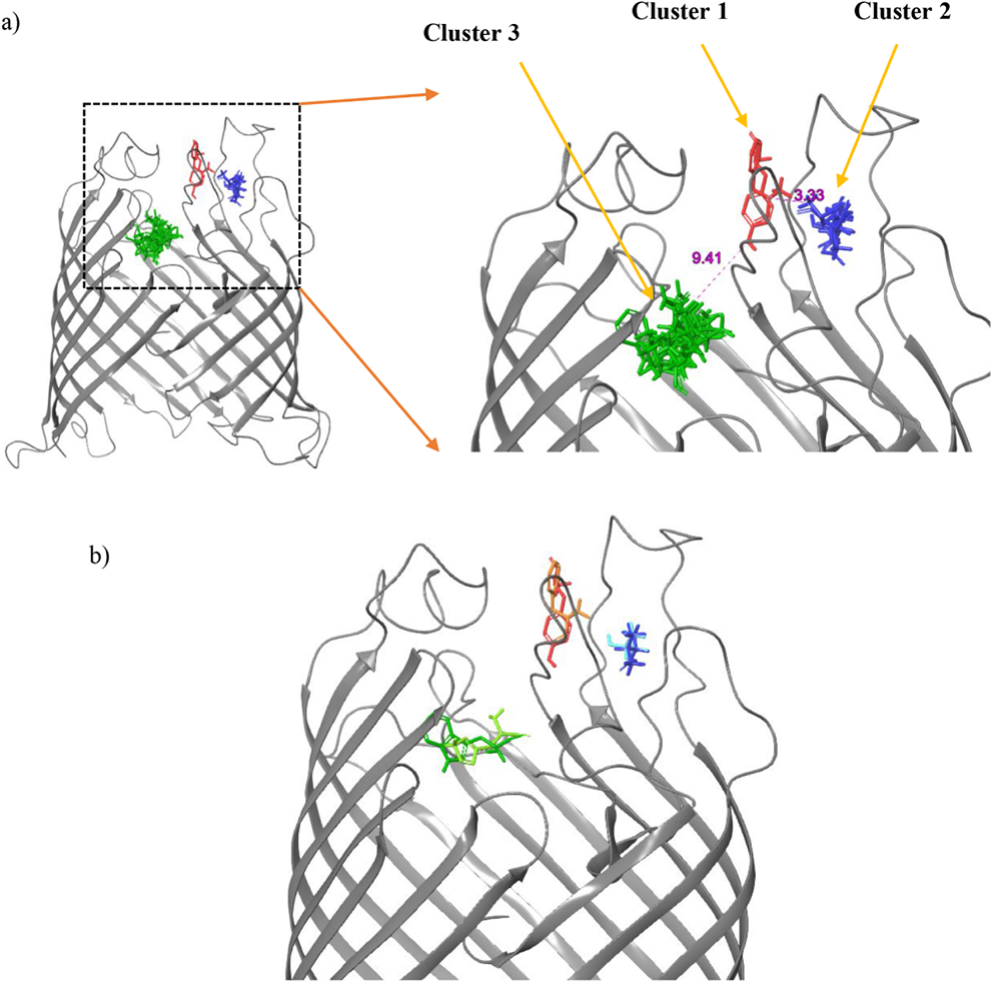
(a) Main amino acid binding sites on BamA (shown in gray ribbons); (b) strongest binding amino acids in each cluster: TYR (red), PHE (orange), VAL (blue), THR (light blue), TRP (green), and HIS (light green).

Figure 1 shows three closely located amino acid clusters that could be identified based on binding site visualization and interaction analysis. The two strongest binding amino acids from each cluster were selected based on docking score and glide energy, parameters indicative of the binding affinity toward the receptor, and were used to develop custom peptide sequences. Table 1 and Figure 1 show that the strongest binding residues for each cluster were PHE and TYR for cluster 1, VAL and THR for cluster 2, and HIS and TRP for cluster 3, respectively. The distances between the clusters were in the range of 3–9 Å, corresponding to one to two amino acids.^53^

These amino acid docking results were used as the foundation for a rational peptide design. A strong-binding amino acid was selected from each cluster, and the three selected amino acids were linked using short peptide segments consisting of 1–2 amino acids, so that the distance between the selected residues would be close to the measured distance between clusters. This resulted in a peptide length of 6–7 amino acids. The linker segments also consisted of strong-binding amino acids to maximize the binding affinity. Overall, 15 peptides were designed using this approach, representing different combinations of strong-binding amino acids in the three clusters.

### Docking of Peptides

The binding of these peptides to BamA was evaluated using docking scores, Glide energies, and MM-GBSA energies, as shown in Table 2.

**Table 2:** Docking Scores, Glide Energies, and MM-GBSA Energies of the Peptides and Their Major Interactions with BamA^a^

Peptide code	Peptide sequence	Docking score (kcal/mol)	Glide energy (kcal/mol)	MM-GBSA energy (kcal/mol)	Residues in BamA forming H bonds
1	FYVTHW	–8.241	–80.677	4.17	ARG675, ASP677, GLY726, ASP728, GLU766
2	FFVVHH	–6.696	–59.849	–13.09	ARG675, HIS722, ASP728
3	FFVTHH	–8.522	–80.824	–24.01	GLU557, ASP677, LEU725, GLY726, ASP728
4	FYVTDHW	–8.517	–69.784	–10.22	LYS558, HIS722, VAL723, LEU725
5	FYVTPHW	–7.772	–68.791	–19.53	LYS558, ARG675, ASP677
6	FFVTDHH	–6.581	–63.364	11.09	ARG675, ASP677
7	FYVTWH	–8.032	–68.674	–13.41	LYS558, ASP728
8	FYVTDWH	–6.982	–55.466	41.08	ASP677, VAL723, GLY726, ASP728
11	WHVTFY	–10.066	–71.401	–30.64	ARG675, HIS722, VAL723
12	WHVTYF	–10.132	–76.399	–11.56	LYS558, ARG675, ASP677, LEU725, GLY726, ASP728
13	HWVTFY	–9.876	–72.787	–9.62	ARG675, ASP677, HIS722
14	HWVTYF	–9.256	–69.179	–13.61	LYS558, ASP677, VAL723, LEU725
15	HHVVFF	–7.620	–51.820	22.48	ARG675, VAL723, ASN733
16	WHVTDYF	–9.677	–74.483	–12.80	ASP677, VAL723, LEU725, GLY726
17	WHTVYF	–8.826	–82.664	–1.67	ASP677, VAL723, GLY726, ASN733
Control	MRL-494 (Control)	–6.115	–54.285	–32.22	ARG675, ASP677, GLY726, ASP728, ASP768

a All energy values are average values based on the three strongest binding poses.

As observed in Table 2, multiple peptides showed docking scores lower than −8.5 kcal/mol and Glide energies lower than −75 kcal/mol, which suggest strong binding with the receptor.^7^ The docking scores and Glide energies of most peptides were lower than those of MRL-494, which has been identified as a BamA inhibitor in multiple Gram-negative bacterial species.^54^ While MM-GBSA energies showed greater variation, the peptides with the lowest docking scores and Glide energies exhibited negative MM-GBSA free energies, indicating thermodynamically favorable binding.^55^ Peptide 3, Peptide 11, and Peptide 12 could be identified as the most promising peptides considering all three parameters.

Interaction analysis revealed that most of the peptides and MRL-494 formed H-bond interactions with several critical residues in BamA. ASP677 was the most crucial residue, forming H-bonds with 13 peptides and MRL-494. Other residues forming hydrogen bonds with most compounds included LYS558, ARG675, VAL723, and LEU725. It could be observed that the majority of the peptides that showed low docking scores or MM-GBSA energies formed at least 5 H-bond interactions with BamA.

Visualization of the binding poses of selected peptides (Figure 2) revealed that all peptides with a strong affinity bound to the same region of BamA, clustering primarily around the extracellular loops at the top of the β-barrel domain—a critical interface for outer membrane protein (OMP) insertion and folding via the BAM complex.^39^ This region has also been used as a target site for other antimicrobial molecules due to its ability to access BamA without having to overcome membrane permeability barriers.^56,57^ Therefore, the binding conformations suggest that the peptides can inhibit BamA by blocking a crucial region of the β-barrel.

**Figure 2:**
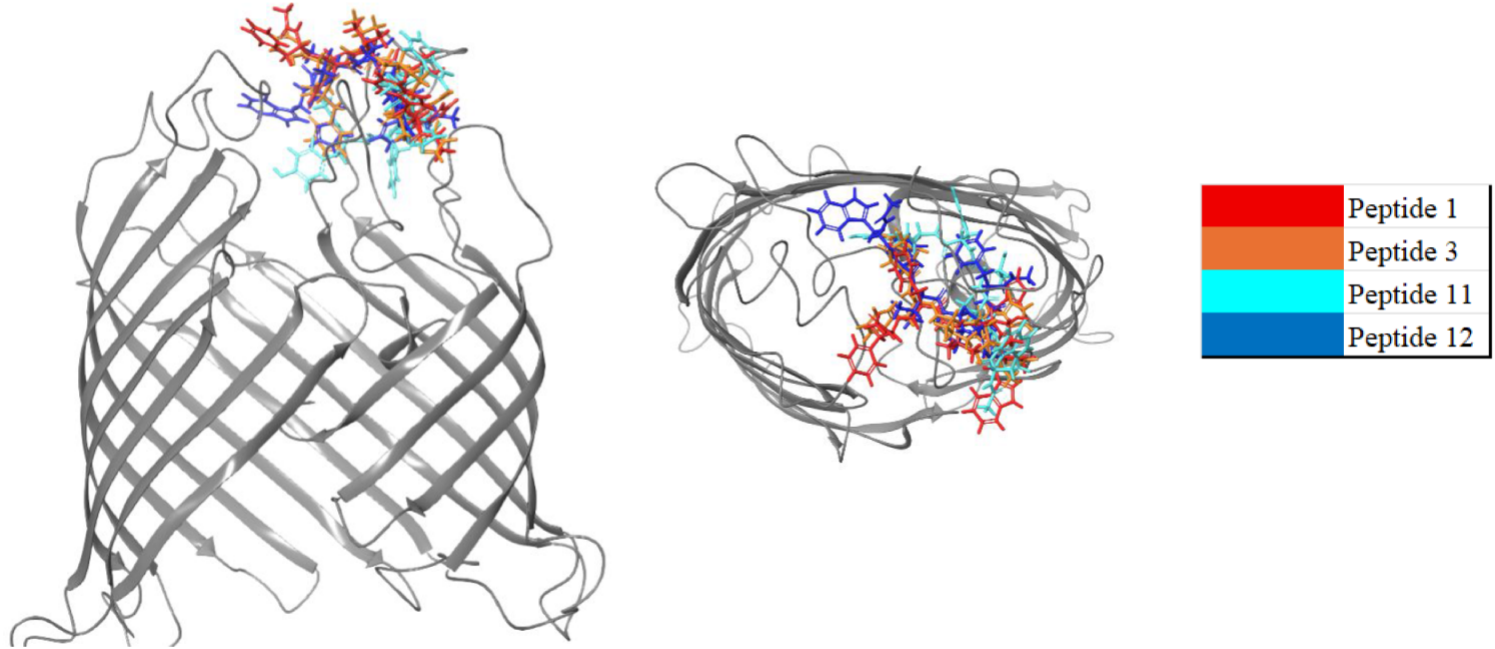
Binding of selected peptides to BamA (shown in gray ribbons). All peptides with a strong affinity toward BamA are bound to the same region.

Strong binding, as indicated by more negative docking scores and MM-GBSA energies, is generally associated with an increased likelihood of interacting with and inhibiting the receptors. We screened four peptides—Peptide 1, Peptide 3, Peptide 11, and Peptide 12—for molecular dynamics and experimental validation based on MM-GBSA energy, docking results, the number of H-bond interactions, and physicochemical features. All the screened peptides demonstrated highly negative docking scores, and Peptides 3, 11, and 12 also showed negative MM-GBSA energies, suggesting thermodynamically favorable binding. Peptide 1 was selected despite a positive MM-GBSA energy based on a highly negative docking score and glide energy, along with the large number of H-bond interactions. The selected set of peptides included two peptides with PHE/TYR at the N-terminus and two peptides with HIS/TRP at the N-terminus to cover a wider selection of physicochemical characteristics.

### Molecular Dynamics Simulations

Molecular dynamics simulations with a duration of 100 ns were conducted to evaluate the structural and conformational changes during peptide binding. Visual observation of trajectories revealed that Peptide 1 demonstrated high stability during the simulation, while Peptide 3, Peptide 11, and Peptide 12 exhibited some fluctuations in binding conformation (trajectory videos are included under Supporting Information). The stability of peptide binding was evaluated based on Root Mean Square Deviation (RMSD) and Root Mean Square Fluctuation (RMSF) analysis. Mean RMSD and RMSF values for BamA, when complexed with each peptide, are given in Table 3, and RMSD and RMSF plots for the α-carbon atoms in BamA–peptide complexes are depicted in Figure 3. Detailed MD reports for each molecule are included in the Supporting Information.

**Table 3:** Mean α-Carbon RMSD and RMSF for BamA–Peptide Complexes

Compound	α-Carbon RMSD (Å)	α-Carbon RMSF (Å)
Free protein	3.310 ± 0.517	1.295 ± 0.701
Peptide 1	2.898 ± 0.251	1.115 ± 0.595
Peptide 3	3.260 ± 0.556	1.250 ± 0.699
Peptide 11	2.903 ± 0.399	1.117 ± 0.522
Peptide 12	3.602 ± 0.486	1.226 ± 0.724
MRL-494 (control)	4.872 ± 0.700	1.585 ± 0.879

**Figure 3:**
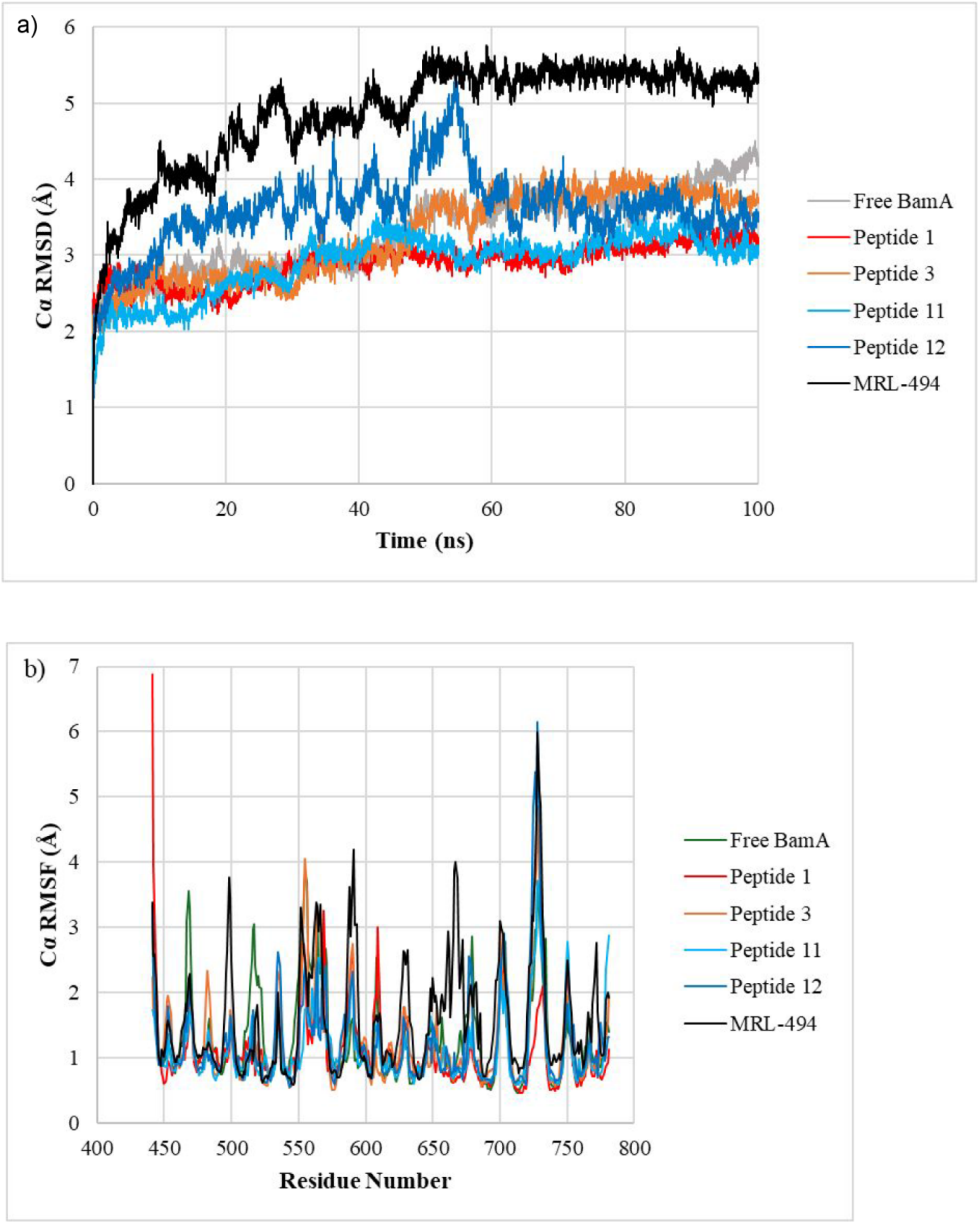
(a) Cα RMSD and (b) per-residue Cα RMSF plots for BamA complexed with peptides during 100 ns of MD simulation.

Table 3 demonstrates that BamA complexes of all peptides have shown lower average RMSD and RMSF compared to the positive control MRL-494, suggesting stable binding. RMSD plots (Figure 3a) show that the BamA complexes of all peptides remain relatively stable after 60 ns of simulation. Peptide 11 showed the lowest RMSD among these compounds, with an average RMSD of 2.903 ± 0.399 Å.

RMSF values were used to determine the stability and flexibility of individual residues during the MD simulation. As shown in Table 3, the binding of all peptides caused a reduction in average RMSF compared to unbound BamA protein, suggesting enhanced structural rigidity upon complex formation. The regions in BamA that have demonstrated a significant decrease in RMSF are 509–524, 556–560, and 674–680. Among these regions, 556–560 and 674–680 contain several key residues that have formed H-bonds with multiple peptides. Therefore, RMSF results indicate strong and stable binding of peptides to the ligands in those regions. Overall, RMSD and RMSF results suggest that all four selected peptides show stable binding to the BamA protein.

The behavior of BamA–peptide complexes during MD simulations was further analyzed by using interactions between BamA and the peptides (Figure 4). Hydrogen bonds, water bridges, and hydrophobic interactions were the main types of interactions between BamA and peptides. The residues that have formed strong interactions with most of the peptides are GLU557, ASP677, and LEU725. These three residues formed H-bonds with most of the peptides during initial docking, suggesting that the peptides have generally stayed stable near their initial binding site. Peptide 1 formed H-bond interactions with multiple residues, including GLU557, GLY726, and ALA727, with water bridges at GLU557, ASP677, and ASP728. For Peptide 3, water bridges were the most common interaction type, while H-bonds were also present at multiple residues, including GLU557, ASP677, and ASP728. Peptide 11 had the most number of contacts with BamA, forming H-bonds, water bridges, and hydrophobic interactions with many residues. While the interaction fraction for individual residues was lower than the other peptides, the greater number of interactions suggests that Peptide 11 binds tightly to BamA. Peptide 12 demonstrated strong hydrogen bonding with VAL723 and LEU725 residues. Overall, the results suggest that all peptides formed strong interactions with BamA during MD simulations.

**Figure 4:**
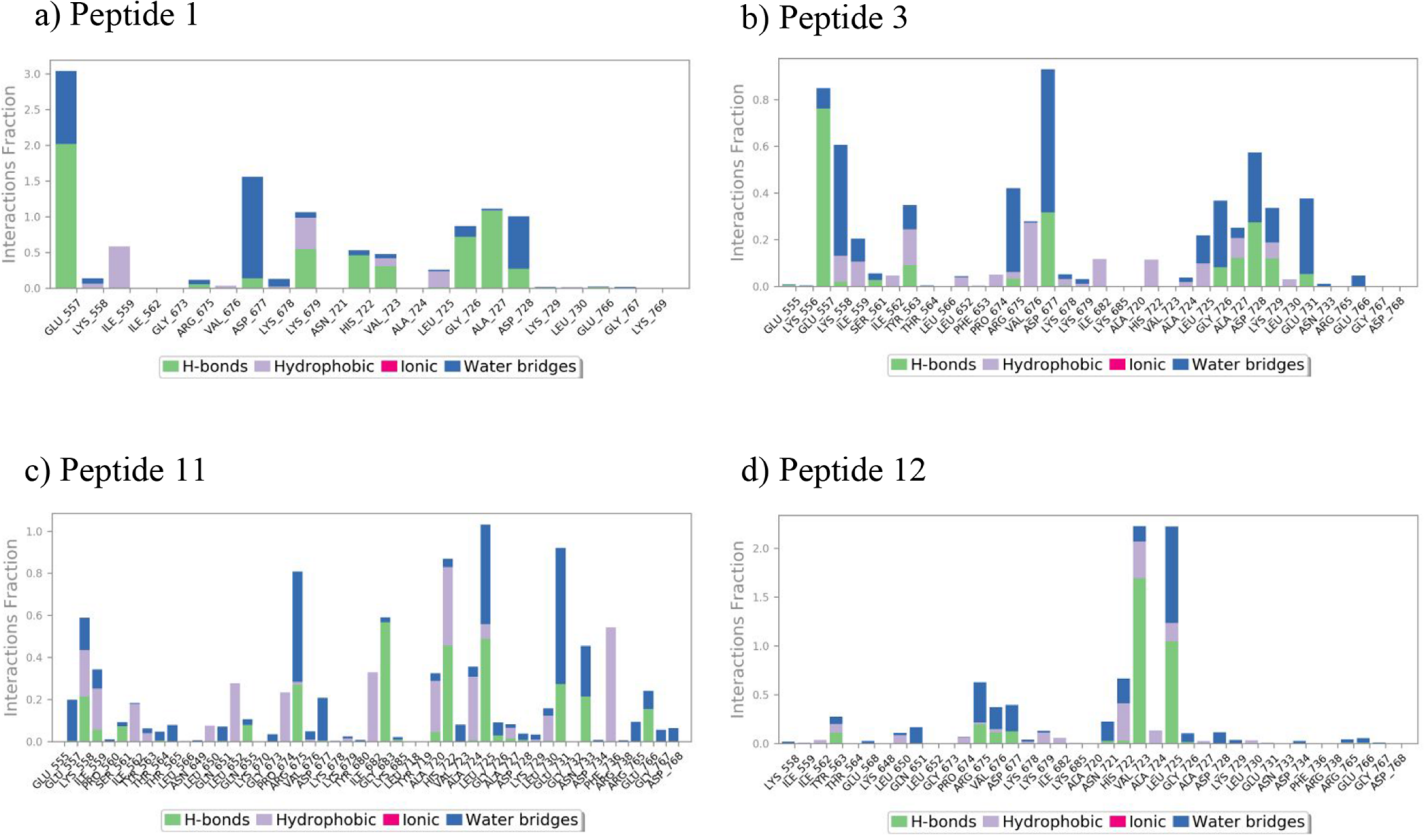
Protein–ligand contacts of (a) Peptide 1, (b) Peptide 3, (c) Peptide 11, and (d) Peptide 12 with BamA. Interactions of each residue of BamA with the peptides are illustrated, along with the fraction of interaction for each interaction type.

### Trajectory MM-GBSA Analysis

The strength of binding of peptides to BamA during the dynamic simulations was evaluated based on MM-GBSA binding free energy during the last 50 ns. The total binding free energy and contributing energy components for each peptide are summarized in Table 4.

**Table 4:** MM-GBSA Binding Free Energy Analysis for the Last 50 ns of the MD Trajectories

Compound	Δ*G* Bind Overall (kcal/mol)	Δ*G* Coulomb (kcal/mol)	Δ*G* Covalent (kcal/mol)	Δ*G* H-bond (kcal/mol)	Δ*G* van der Waals (kcal/mol)	Δ*G* lipophilic (kcal/mol)	Δ*G* Solvation (kcal/mol)	Ligand strain (kcal/mol)
Peptide 1	–51.57 ± 7.42	–15.47 ± 9.70	0.82 ± 4.17	–1.70 ± 0.55	–45.18 ± 4.57	–14.75 ± 2.92	24.29 ± 7.60	11.40 ± 5.08
Peptide 3	–44.83 ± 6.99	–12.36 ± 10.65	0.11 ± 3.44	–2.67 ± 0.69	–47.26 ± 3.83	–16.51 ± 3.14	34.79 ± 7.36	10.93 ± 3.51
Peptide 11	–79.20 ± 6.53	–20.28 ± 3.50	3.62 ± 1.57	–1.66 ± 0.46	–65.18 ± 4.14	–26.52 ± 2.03	31.95 ± 3.22	8.85 ± 3.17
Peptide 12	–43.65 ± 10.12	–14.08 ± 9.45	–0.90 ± 5.16	–1.03 ± 0.82	–35.45 ± 3.64	–9.67 ± 4.28	17.36 ± 4.08	15.89 ± 9.12
MRL-494 (control)	–37.79 ± 3.94	–486.30 ± 79.59	5.70 ± 2.78	–2.21 ± 0.46	–29.48 ± 4.22	–8.44 ± 1.61	482.94 ± 78.29	6.25 ± 2.46

According to Table 4, all the peptides showed more negative Δ*G*_Bind Overall_ than the positive control MRL-494, indicating strong affinity to BamA. Peptide 11 showed the most favorable overall binding free energy of −79.20 ± 6.53 kcal/mol, which indicates very strong and stable binding to BamA.^58^ The other three peptides also demonstrated Δ*G*_Bind Overall_ values of less than −40 kcal/mol, indicating moderately strong binding. Analysis of individual energy components revealed that Δ*G*_van der Waals_ made the largest contribution to the binding energy of all peptides, with Δ*G*_Coulomb_ and Δ*G*_lipophilic_ also making significant contributions. Notably, Peptide 11 also had the most favorable component energies across all categories, further supporting its superior binding profile. It also demonstrated the lowest ligand strain energy among the peptides, indicating that it binds in a conformation that has better structural compatibility with the binding site.^59–61^ Collectively, these results suggest that Peptide 11 has the highest binding potential and conformational suitability to the BamA protein, while the other three peptides also demonstrate strong and stable binding.

### BioLayer Interferometry

BioLayer Interferometry (BLI) was used to determine the binding kinetics of the screened peptides on BamA. Among the four peptides, Peptide 3 did not give a detectable response, indicating it does not have a good affinity toward BamA. Average values of binding parameters for the other three peptides, based on three replicates, are given in Table 5, and BLI graphs for the strongest binding peptides are shown in Figure 5.

**Table 5:** Binding Parameters of the Screened Peptides on BamA Determined Using BioLayer Interferometry^a^

Peptide	Association rate (*K*_a_) (1/M s)	Dissociation rate (*K*_d_) (1/s)	Affinity constant (*K*_D_) (M)
Peptide 1	(3.254 ± 2.998) × 10^2^	<10^–7^	(6.024 ± 3.740) × 10^–10^
Peptide 11	(3.610 ± 3.422) × 10^2^	<10^–7^	(2.380 ± 1.834) × 10^–10^
Peptide 12	(1.716 ± 2.024) × 10^3^	(5.925 ± 5.565) × 10^–3^	(1.653 ± 1.922) × 10^–5^
MRL-494	(1.840 ± 0.501) × 10^4^	(1.272 ± 0.989) × 10^–4^	(7.200 ± 5.111) × 10^–8^

a Each value is expressed as the mean ± standard deviation based on three replicates.

**Figure 5:**
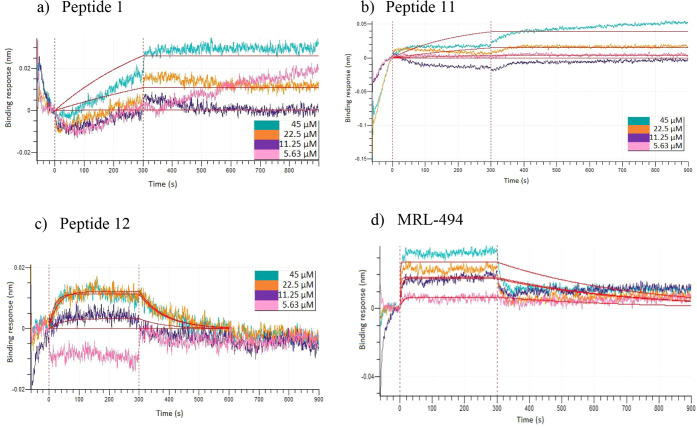
Association and dissociation curves for the binding of (a) Peptide 1, (b) Peptide 11, and (c) Peptide 12, and (d) MRL-494 on BamA. Association occurs during the initial 300 s, and dissociation occurs during the final 600 s.

Based on the BLI results presented in Table 5, it can be observed that Peptide 1 and Peptide 11 bind exceptionally tightly to BamA, characterized by dissociation rates of less than 10^–7^ s^–1^, and affinity constants in the picomolar range. These two peptides demonstrated stronger binding affinity than the positive control MRL-494, which had an affinity constant of (7.2 ± 5.1) × 10^–8^ M. These results agree with the molecular dynamics results, where these two peptides showed minimal fluctuations (Table 3 and Figure 3), indicating stable binding. The slow dissociation of these two peptides can also be observed in the BLI sensorgrams (Figure 5), which depict nearly horizontal dissociation curves. The dissociation curves and kinetic parameters for these two peptides are also consistent with other strong interactions reported in literature.^62–66^ In comparison, the sensorgrams of Peptide 12 and MRL-494 show much faster dissociation, which agrees with the affinity parameters. While Peptide 12 showed weaker affinity, it had the fastest association rate among peptides and displayed association and dissociation behavior similar to that of MRL-494. Thus, these three peptides are identified as potential candidates for BamA inhibition based on kinetic parameters.

### In Vitro Efficacy Testing

Since CLas is unculturable, in vitro efficacy testing was conducted using the surrogate bacterium *Rhizobium grahamii*. To justify this choice, we compared the sequence and structure of CLas and *R. grahamii* BamA proteins. The BamA protein of *R. grahamii* demonstrated 39% sequence identity to CLas BamA protein (Figure S5), with an *E*-value of 0, indicating that the BamA structures are significantly homologous.^67,68^ It also demonstrated 42.5% sequence identity in the extracellular and transmembrane regions that are important for peptide binding, which was one of the highest sequence identities among culturable candidates. Structural alignment of CLas and *R. grahamii* BamA proteins using Schrodinger protein structure alignment tool revealed a low RMSD of 1.949 Å, indicating high structural similarity.^69^ Given this level of sequence and structural conservation in the predicted binding regions, peptides that inhibit *R. grahamii* BamA are expected to have a similar impact on CLas BamA.

The inhibition of *R. grahamii* by each peptide was analyzed at four different dosages (10, 25, 50, and 100 μg/mL), using Kanamycin and MRL-494 (100 μg/mL) as positive controls. The activity of peptides against *E. coli* was evaluated to determine the target specificity of peptides. The results of in vitro efficacy testing are presented in Figure 6, and the time-course microbial growth curves are presented in Figure S2.

**Figure 6:**
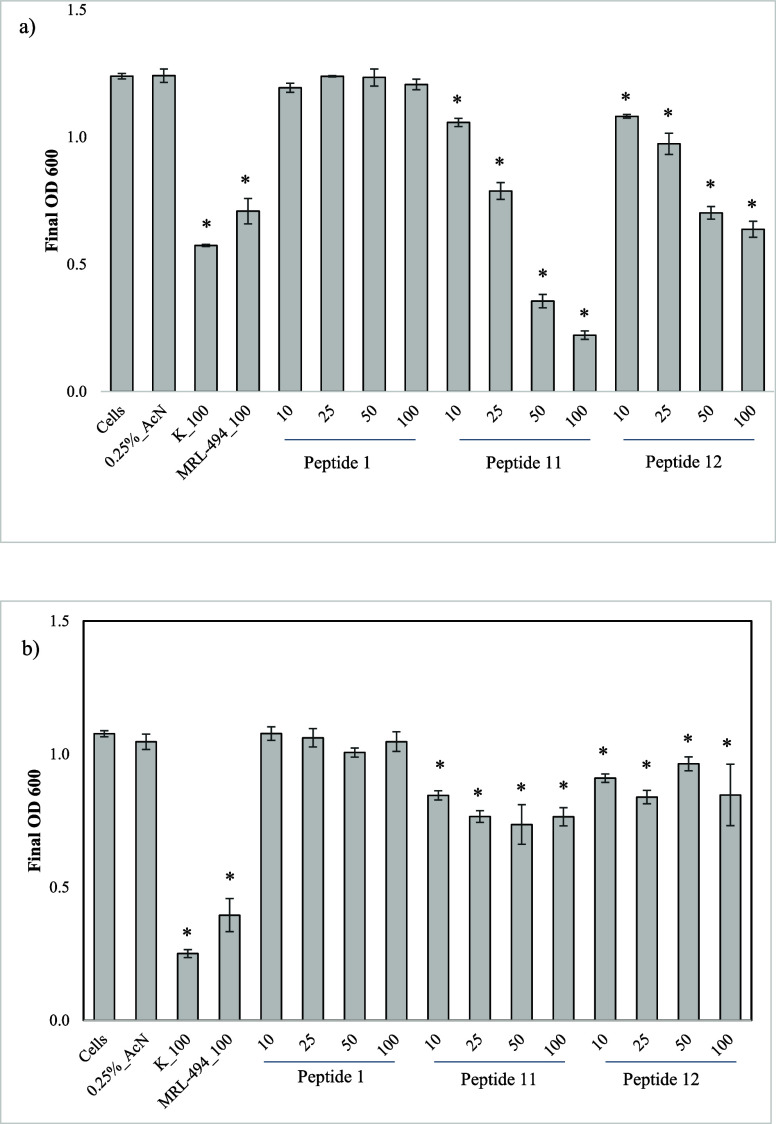
Efficacy of binders 1 to 19 on (a) *R. grahamii* and (b) *E. coli*. Asterisks (*) represent treatments that were significantly different (*p* ≤ 0.05) compared with untreated or solvent-alone controls. Error bars represent the ±standard error of the mean (*n* = 3). The peptide dosages shown are in μg/mL.

As depicted in Figure 6, Peptide 11 and Peptide 12 showed statistically significant inhibition of *R. grahamii* at all tested dosages. Peptide 11 has demonstrated the most promising results among these two peptides, surpassing Kanamycin’s inhibitory effects on *R. grahamii*. Peptides 11 and 12 have also shown significant inhibition of *E. coli*. However, compared to Kanamycin and MRL-494, these two peptides have shown a lower level of inhibition of *E. coli*. None of the other peptides have inhibited *R. grahamii* or *E. coli*, suggesting only Peptides 11 and 12 possess antimicrobial properties.

Overall, in vitro analysis indicates that Peptides 11 and 12 cause greater inhibition of the CLas surrogate *R. grahamii* compared to *E. coli*, consistent with their designed selectivity toward CLas BamA. However, a broader assessment of their antimicrobial activity against other bacteria in the rhizosphere is required to fully confirm the specificity, which will be a focus of future studies. Based on current results, Peptides 11 and 12 can be identified as promising candidates with narrower activity profiles than broad-spectrum antibiotics such as Kanamycin.

## Discussion

Due to the unculturable nature of CLas bacterium, in silico molecular modeling is considered a fast and practical approach to screen drugs against citrus greening. In this study, we present a novel peptide design technique, which is based on identifying and linking the amino acids with the highest affinity toward the target region in BamA. Using this approach, we constructed peptide sequences with strong binding affinities toward BamA. Initial docking results revealed several peptides with very high affinity toward BamA, with docking scores less than −8.5 kcal/mol and Glide energies less than −75 kcal/mol. Molecular dynamics simulations were conducted to gain a dynamic perspective on peptide binding and assess the stability and conformational changes over time. The analysis of RMSD and RMSF values provided evidence of stable binding for several peptides, particularly Peptide 1, Peptide 3, Peptide 11, and Peptide 12. These peptides demonstrated minimal fluctuations in binding conformation, suggesting robust interactions with BamA. Peptide 11 also demonstrated highly negative trajectory MM-GBSA energies, indicating very strong binding. Experimental validation of binding affinity was done using BioLayer Interferometry, based on which three peptides were identified as having a high affinity toward BamA, two of them demonstrating even higher affinity than the known BamA inhibitor MRL-494.

In vitro assays conducted using *R. grahamii* revealed that Peptides 11 and 12 showed selective and effective inhibition of *R. grahamii*, revealing that these peptides will be most promising for CLas inhibition. Analysis of docking interactions between the peptides and BamA provides some insights into why Peptides 11 and 12 inhibit *R. grahamii*. According to Table 2, these two peptides showed the most negative docking scores among all peptides, with docking scores less than −10 kcal/mol, indicating very tight initial binding. As observed in Figure S1, these two peptides, particularly Peptide 11, formed both hydrophobic and polar/H-bond interactions with BamA. In Peptide 11, the TYR residue interacted with hydrophobic LEU650, LEU652, LEU718, and TYR719 residues, while the HIS residue formed strong H-bonds and polar interactions in the ARG675-LYS679 region, similar to the positive control MRL-494. The stability of these interactions was confirmed in molecular dynamics, where Peptide 11 exhibited stable interactions with multiple regions in BamA, as illustrated in Figure 4. We believe that the antimicrobial activity of Peptide 11 could be attributed to the tight binding to both hydrophobic and hydrophilic regions in BamA, which could lead to effective BamA inhibition. While Peptide 12 demonstrated fewer interactions compared to Peptide 11, its high sequence similarity to Peptide 11 could have contributed to its antimicrobial activity by having similar critical physicochemical properties, such as charge and hydropathicity. Both peptides had a net charge of zero and a grand average of hydropathicity of 0.150, suggesting an overall hydrophobic nature and an ability to interact with cell membranes.^70^ Also, while the binding stability of Peptide 12 was lower than that of Peptide 11, it demonstrated highly negative docking scores in molecular docking and fast association in BLI, suggesting that its strong initial binding may be sufficient to cause an inhibitory effect on the CLas surrogate.

Future directions for this study include evaluating the in-plant efficacy of these peptides and determining the optimal way to use these peptides against citrus greening disease, either as direct inhibitors or as targeting moieties in smart-targeting AMPs. Additionally, elucidating the mechanism of action of these peptides and evaluating the contribution of physicochemical features to the antimicrobial activity can help refine the peptide design workflow, which will be the focus of our future work.

Overall, this peptide design approach represents a mechanistically distinct and complementary strategy to both traditional and modern AI-based peptide design approaches through its integration of residue–residue interaction dynamics, structural adaptability, and computational efficiency. Conventional AMP design approaches rely on sequence comparison with known AMPs, motif-based heuristics, and genome mining.^71–73^ Modern AI-based approaches, such as ProteinMPNN, LigandMPNN, and Boltz, rely on machine learning (ML) models trained on existing protein sequences and structures for designing peptide binders.^74–77^ In contrast, this study employs a residue-level docking approach to optimize receptor–ligand interactions, which helps to identify and assemble peptide fragments based on their actual binding contributions rather than statistical correlations or rigid templates, offering greater mechanistic transparency than ML-based approaches. Additionally, this approach enables the mapping of multiple binding sites, which facilitates the rational design of peptides targeting undruggable sites that do not possess clearly defined binding pockets and are difficult to target using traditional or ML-based approaches due to the lack of structural data.^78,79^ This workflow will also enable rapid redesign of peptides in response to resistance-causing mutations by analyzing the impact of mutations on residue-level interactions. Thus, this approach has great potential for rapid and rational design of antimicrobial peptides with high affinity and superior specificity toward target proteins in pathogenic microorganisms, with a unique approach than current machine learning or motif-based approaches.

## Methods

### Development of BamA Structure

The same BamA homology model used for the previous work regarding STAMPs was used for this study.^80^ Homology models were initially built using SWISS-MODEL server, based on the CLas BamA sequence obtained from UniProt database (UniProtKB ID: Q32TE9), using 15 structures with >25% sequence similarity to CLas BamA as templates. The best model was selected based on GMQE (Global Model Quality Estimate) and QMEAN scores. The selected model used the Bam complex of *E. coli* (sequence identity of 25%) and had a GMQE of 0.65, the highest among the models, and a favorable QMEAN score of −3.03. The quality of the homology model was evaluated using the Ramachandran plot,^81^ which revealed 88% of the residues in the most favorable regions and 10% of the residues in additionally allowed regions. Additionally, ERRAT analysis^82^ yielded an overall quality factor of 88.2%, with the majority of residues near the binding site demonstrating low error values. The Ramachandran plot and ERRAT plots are included in Figures S3 and S4, respectively.

### Development of Modified Amino Acid Structures

Structures of the amino acids were obtained from the ZINC15 database. The terminal carboxyl groups of amino acids were replaced with less reactive carbonyl groups to reduce terminal reactivity and emulate the behavior of amino acids in peptide bonds. Amino acid structures were modified using Schrodinger maestro and optimized using protein preparation wizard. The structures were minimized using the OPLS_2005 force field to ensure that they would have a stable low-energy conformation.

### Docking of Amino Acids and Peptides

Amino acids and peptides were docked on the BamA protein using Schrodinger Glide standard precision docking. The top part of the barrel was selected as the binding domain due to higher accessibility from the extracellular space. The docking grid was generated with ASP677 and LEU725 as the centroid, since those regions formed strong interactions with AMPs in our previous studies,^80^ and the grid size was kept at 36 Å. The protein and all ligands were optimized and minimized using the Schrodinger protein preparation tool prior to docking.

### Molecular Dynamics Simulations

Molecular dynamics simulations for protein–ligand complexes were conducted using Schrödinger Desmond. Protein–ligand complexes resulting from Glide were set up by using the system builder tool. The solvation process utilized the SPC solvent model and OPLS_2005 force field, and neutralization was achieved by adding Na^+^ or Cl^–^ ions. The POPC membrane model with beta-sheets as transmembrane atoms was used to place the membrane. Molecular dynamics simulations were run for 100 ns with a recording interval of 20 ps under an *NPT* ensemble at 300 K and 1.01325 bar. Before the simulation, the system was relaxed by using the default relaxation protocol in Desmond. Postsimulation analysis was generated using Simulation Interaction Diagram and Simulation Event Analysis tools in Desmond. Root Mean Square Deviation (RMSD) was used to evaluate the structural stability of protein–ligand complexes during the simulation duration, and Root Mean Square Fluctuation (RMSF) was used to identify fluctuations within different regions of the protein.

### MM-GBSA Energy Calculations

Molecular mechanics-generalized Born surface area (MM-GBSA) energy was used to measure the binding free energy of peptides to BamA. MM-GBSA energies based on initial docking poses were calculated using the Prime MM-GBSA tool based on the following equation.

$$\Delta G\left(\mathrm{bind}\right)=E_{\mathrm{complex}\left(\mathrm{minimized}\right)}-\left(E_{\mathrm{ligand}\left(\mathrm{minimized}\right)}+E_{\mathrm{receptor}\left(\mathrm{minimized}\right)}\right)$$

The embedded script thermal_mmgbsa.py was used to calculate the MM-GBSA energies for Desmond trajectories. These calculations were based on 500 frames during the last 50 ns of the simulation, with an interval of 100 ps between frames.

### Materials

CLas BamA protein was expressed in vitro by GenScript using peptide sequences that have been previously published.^83^ Designed peptides were custom-synthesized by GenScript based on sequences provided by the authors. Tris buffer (pH 6.5) was procured from VWR, USA, while APS biosensors and Sartorius kinetic buffer were purchased from Sartorius.

### BioLayer Interferometry

ForteBio Sartorius Octet system was utilized to evaluate the binding kinetics of the peptides on BamA. Experiments were conducted in advanced kinetics mode using aminopropylsilane (APS) biosensors. BamA protein was dissolved in pH 6.5 Tris buffer, while all other solutions were prepared using 1× Sartorius kinetics buffer. Initially, the biosensors were hydrated for 10 min in pH 6.5 Tris buffer, followed by a 60 s baseline step. BamA protein was loaded on the biosensors by immersing the biosensors for 300 s in a 5 mg/L protein solution, followed by another 60 s baseline step in 1× kinetics buffer to stabilize the protein on the sensor. Association and dissociation steps were conducted for 300 and 600 s, respectively. For each compound, association and dissociation experiments were performed at four different concentrations at 2-fold serial dilutions, and a zero-concentration sample was used as the baseline reference. Each biosensor was used only once, and no regeneration was done. A set of reference sensors that were not loaded with BamA was employed to account for nonspecific binding,^84^ and the kinetic analysis was conducted based on the difference in binding response between loaded and reference sensors.

The Octet Analysis software was used to calculate association and dissociation rates and affinity constants based on the binding curves, utilizing a 1:1 global-fitting model. All experiments were performed in triplicate, and the binding parameters were expressed as the mean ± standard deviation.

### In Vitro Efficacy Assays

The peptides were synthesized by Genscript, USA, based on sequences provided by the authors. Starter cultures of *Rhizobium grahamii* and *Escherichia coli* were prepared in approximately 3 mL of TY medium (5 g Bacto Tryptone, 3 g yeast extract, 1.3 g CaCl_2_·6H_2_O, deionized water to 1 L; add 15 g/L for solid media) to an optical density (OD) of 0.5–0.7. For the efficacy assays, cells were subsequently resuspended in acetonitrile to a final OD of 0.1 in 500 μL of YM media (3.0 g of yeast extract, 3.0 g of malt extract, 10.0 g of dextrose, 5.0 g of peptone; adjust pH to 6.2, deionized water to 1 L) supplemented with the respective peptides at concentrations of 0, 10, 25, 50, or 100 μg/mL, alongside an antibiotic (Kanamycin 100 μg/mL), MRL-494 (100 μg/mL), and untreated and solvent (1.25% v/v) controls. All assays were conducted in triplicates in a transparent 96-well U-bottom multiwell plate with a lid to prevent evaporation (Corning Falcon, Fisher Scientific, Hampton, NH). The assay plates were placed in a SYNERGY H1Microplate reader equipped with Gen5 3.0 software, manufactured by Agilent, Santa Clara, CA. The plates were incubated at 28 °C with continuous shaking. OD_600_ readings were measured every hour until untreated controls reached an OD_600_ of approximately 1.

## Supplementary Material



## Data Availability

All relevant data are included in the manuscript and Supporting Information. Any additional information is available from the authors upon request.

## Notes

Ligand interaction diagrams (Figure S1); microbial growth curves (Figure S2); protein structure validation results (Figures S3 and S4); sequence comparison for *R. grahamii* and CLas (Figure S5); and trajectory videos for MD simulations (https://zenodo.org/records/15858791) (PDF)
