# Supplementary material for: Design of Highly Specific Antimicrobial Peptides Targeting the BamA Protein of Candidatus Liberibacter Asiaticus
Source: ACS Omega. 2026 Feb 2;11(6):10144–55. doi: 10.1021/acsomega.5c11153 (PMC12917781; doi:10.1021/acsomega.5c11153)
Supplement: Supplementary file 1 [file ao5c11153_si_001.pdf]

Design of Highly Specific Antimicrobial Peptides Targeting the BamA protein of *Candidatus* Liberibacter asiaticus

Samavath Mallawarachchi[1], *Sonia Irigoyen*[2], *Kranthi Mandadi*[2,3,4], *James Borneman*[5], *Sandun Fernando*[1*]

[1]Department of Biological and Agricultural Engineering, Texas A&M University, College Station, TX 77843, USA.

[2]Texas A&M AgriLife Research & Extension Center, Texas A&M University System, 2415 E. Highway 83, Weslaco, TX 78596, USA

[3]Department of Plant Pathology and Microbiology, Texas A&M University, College Station, TX 77843, USA

[4]Institute for Advancing Health through Agriculture, Texas A&M AgriLife, College Station, TX 77843, USA

[5]Department of Microbiology & Plant Pathology, University of California Riverside, Riverside, CA 92507, USA

[*]Correspondence: sandun.fernando@ag.tamu.edu

Peptide 1

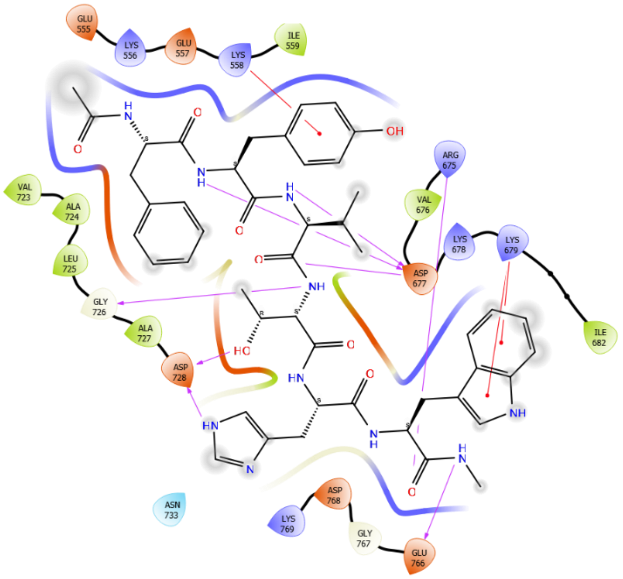

Peptide 3

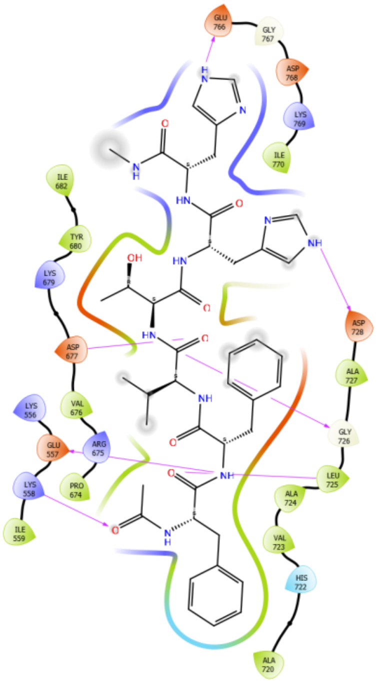

Peptide 11

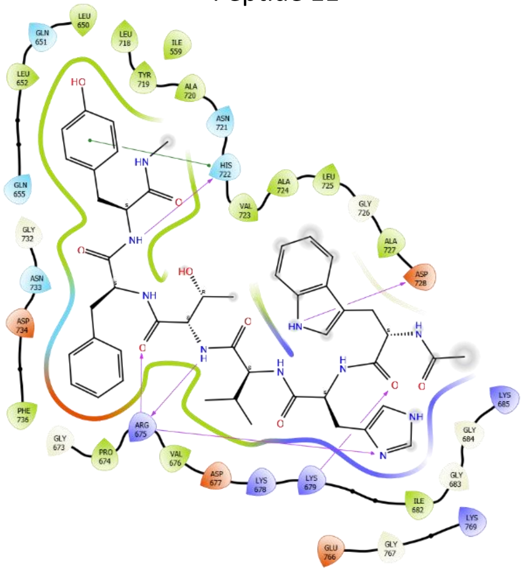

Peptide 12

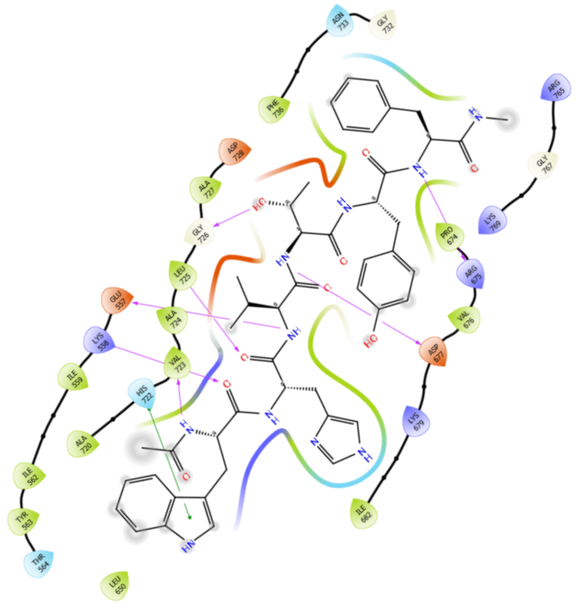

MRL-494
(Positive control)

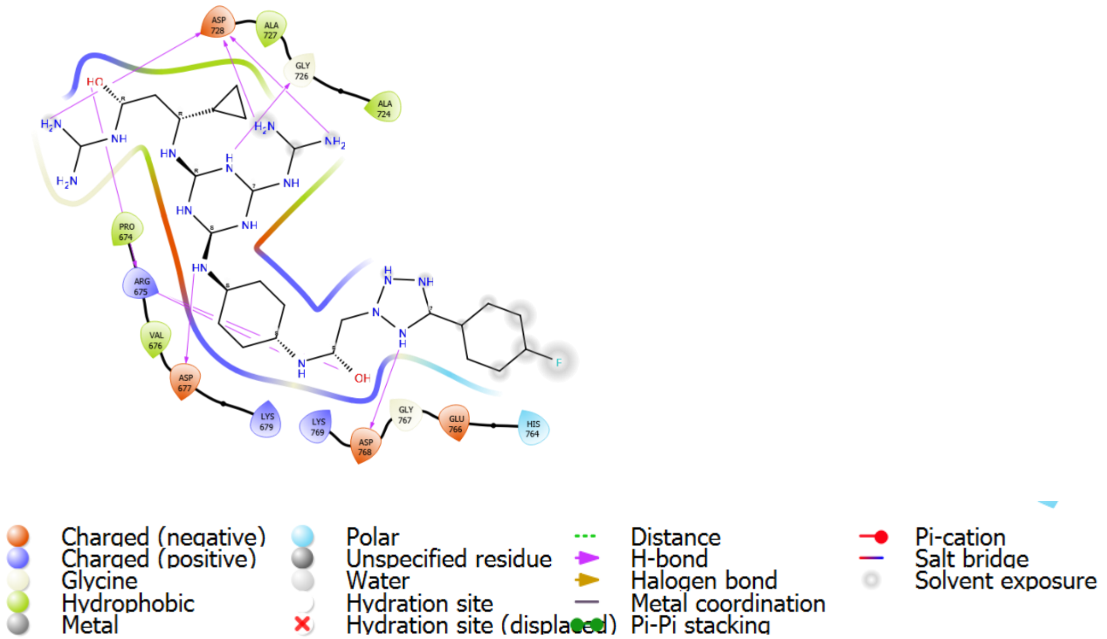

Figure S1 – Ligand Interaction diagrams of the peptides with BamA

a)

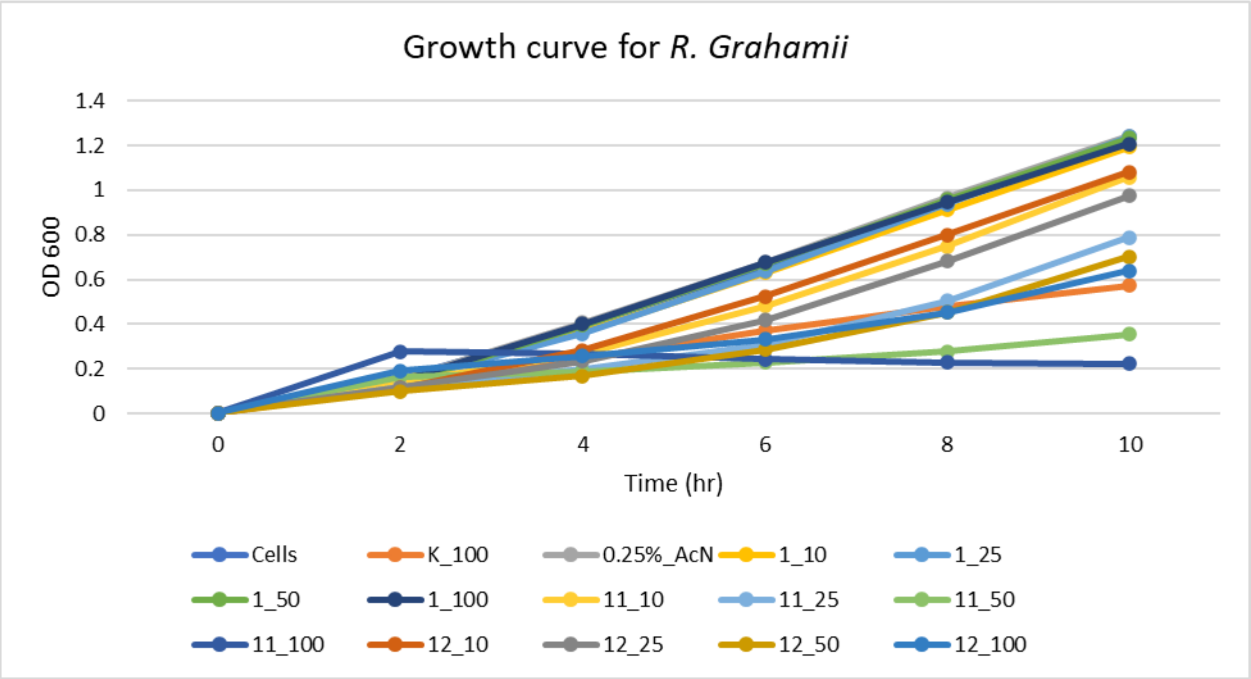

b)

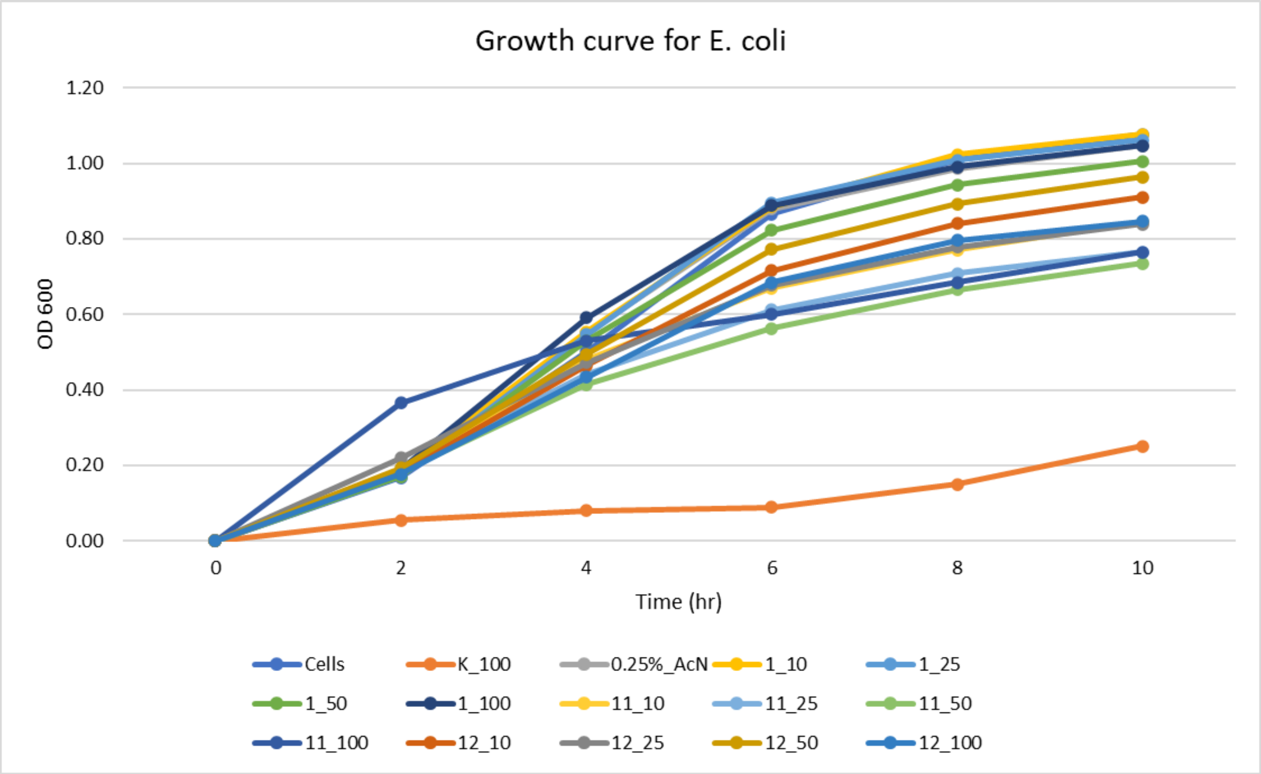

Figure S2 – Microbial growth curves for a) *R. Grahamii* and b) *E. Coli* under different treatments. Treatments are labelled as name_concentration. All values are averaged over three replicates.

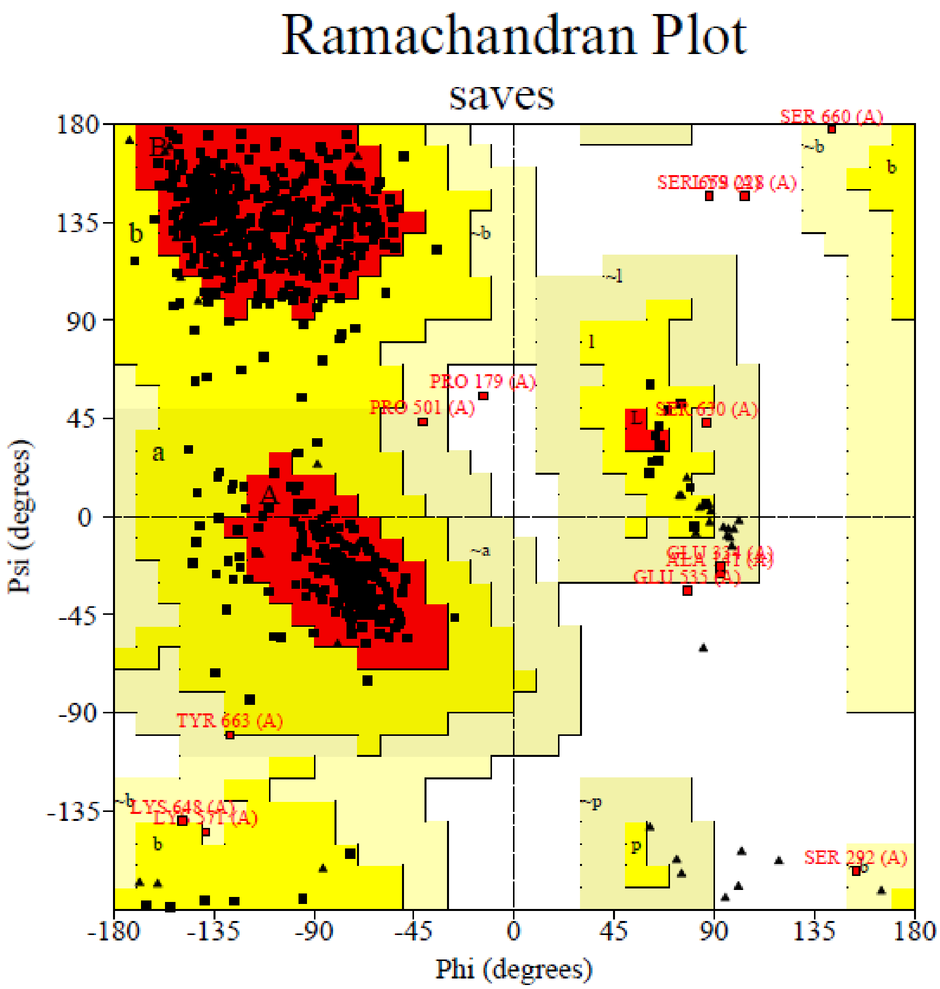

Plot statistics

Residues in most favoured regions [A,B,L]	577	88.0%
Residues in additional allowed regions [a,b,l,p]	68	10.4%
Residues in generously allowed regions [~a,~b,~l,~p]	8	1.2%
Residues in disallowed regions	3	0.5%
	----	------
Number of non-glycine and non-proline residues	656	100.0%
Number of end-residues (excl. Gly and Pro)	2	
Number of glycine residues (shown as triangles)	57	
Number of proline residues	20	

Total number of residues	735	

Figure S3 – Ramachandran Plot for the BamA homology model. 88% of the residues are in the most favorable regions and 10% of the residues are in additionally allowed regions.

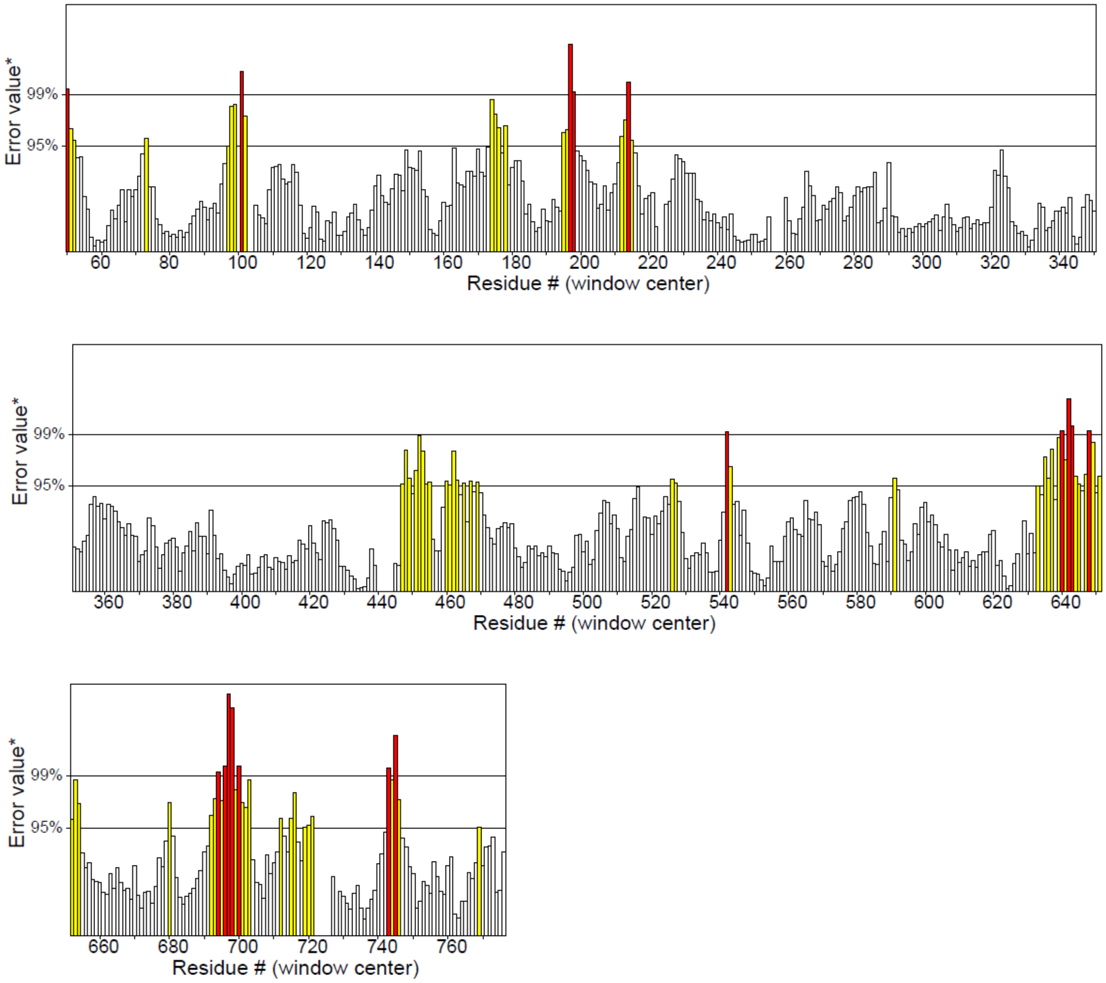

Figure S4 – ERRAT Plots for BamA homology model.

a)

```
Identities:291/745(39%), Positives:447/745(60%), Gaps:9/745(1%)

Query  43   SNTSIVRRIEIRGATNVGKEVILSRIPVVVGQSISDADLDHAVKNIYAMGYFSNVKIKIV  102
            +  ++++R+++RGA+ VG E + S + +  G+S S+ D+D +VK +Y  GYFS+VKI +
Sbjct  36   AEAAVIQRVDVRGASRVGAEAVRSNLTITPGKSFSNTDIDDSVKQLYDTGYFSDVKIAVS  95

Query  103  DSVLIIDLIERKIINHLFFSGNNNLKDDQLKMIVRSRSAAAYDEDTVNADVHNIKQAYAS  162
               L++ + E +++N + F+GN  +KDD+L  +V++ +A  Y E  V AD+  IK AYA+
Sbjct  96   GGTLVVTVQEAQLVNQVVFNGNRKIKDDKLAAVVKTHAAGPYSEAQVQADIQAIKDAYAA  155

Query  163  IGYLNVMVKVQHHSISPTTLNITYVIEEGVKAKINSIRFVGNKNYSHARLERVISIRTSG  222
             G   V V  Q   +    +N+ YV+ EG + KI +I FVGN  YS  RL  VI+ + S
Sbjct  156  TGRSEVEVTTQVVPLGEGRVNLAYVVNEGDRTKIAAINFVGNSAYSSGRLASVINTKRSN  215

Query  223  YFSF-GKTDVYSKERMSFDEEAIRAFYHDRGYAAVK-VSSQVLFDKQKSGYVLIFQIDEG  280
            + SF  + DVYS++++  DEEA+R FY++RGYA  + VSS   FD+  + Y L F I+EG
Sbjct  216  FLSFLTRKDVYSEDKLHADEEALRQFYYNRGYADFRIVSSDAAFDEGTNKYTLTFNIEEG  275

Query  281  EIYTVGNISIQSTLQEIQKKTLLSLIRIRSGNLYNPQEIKESSEKISKYFFSGERPFVRV  340
              Y  G +++QST+Q I    L  L+R   G++YN +E+++S E IS    S   PF RV
Sbjct  276  PRYDFGAVTVQSTVQGIDGAQLQGLVRTHEGDVYNAKEVQKSIEAISDQVASAGYPFARV  335

Query  341  KTRINRDFAKRIVDIEYLIDQGSPLYVKRIEIEENDQSYDSVIRRELELSEGDPINYSMI  400
              R NRD     + +EYL+DQG   YV+RIEI  N ++ D VIRRE ++SEGD  N  MI
Sbjct  336  TPRGNRDLNNNTIGVEYLVDQGERAYVERIEIRGNSRTRDYVIRREFDMSEGDAFNQQMI  395

Query  401  ERAKRRIMATGYFSEVNISQLPANDVSDYVILRVSVKQLSAGSVGIATNYEVDKGMGVEG  460
             +AKRR+ A GYFS VNIS  P +     V++     Q +      A       G+ +E
Sbjct  396  TKAKRRLEALGYFSAVNISTQPGSSPDRVVVVVDVQDQATGSFGIGAGYAAGGDGLLLEA  455

Query  461  HIDDNNFFGQGYRARLAAGFGRHAVQNYTFSVEDPYFLGSPISAGFDLQKLEDGS---LD  517
             +++ NF G+G   +++AG G    + Y  +  +PYFLG  ++ GFD+   E  S    D
Sbjct  456  SVEEKNFLGRGQYIKISAGGGEEGSRTYGLNFTEPYFLGYRLAVGFDINHSETSSNDNYD  515

Query  518  INDESAAVRMIVPITESISTSFKYDLRFLQYGAISEKEKIPSIYTTLIEHGKFSSHSISQ  577
              + +  +R   PITE ++T+F+Y+ + ++Y        + + Y  L+ +  +   S+SQ
Sbjct  516  YEENNVVLRATAPITEDLATTFRYNYKQMKYDPSGSLSDLSTPYQDLVNNSPWVKSSVSQ  575

Query  578  SIIYNTLDNPIVPRKGMLISSSYDYAGFGGDSQYHRIGSRASYFYLLSDDSDIVGSLRFG  637
            ++ YNTLD+ I+PR+G+  S++ + AG GGDSQ+++I  +A Y++LL+DD+DI+GS+
Sbjct  576  TLTYNTLDDMILPREGIYASATQEIAGLGGDSQFYKIYGKARYYHLLADDADIIGSVAGS  635

Query  638  YGCVIPSNKNLQLFDQFSVSSNYYLRGFAYKGIGPRV--DKKYAIGGKIYSSASAAVSFP  695
             G V+    NL +FDQF++ +N  +RGF  KGIGPRV       +GG  Y + SA  +FP
Sbjct  636  AGYVVGFGDNLHVFDQFTL-TNGDIRGFENKGIGPRVGGGPDDPLGGTTYFTVSAEATFP  694

Query  696  MPLVPERAGLRGAFFVDSATLYANHVALGADK-LEGNDSFWRVSTGVEIMWNSPLGMMGV  754
            +P VP   GLRGA F D+ TL+ N V++G  + + G D+  R S GV ++WNSP G + V
Sbjct  695  LPAVPRDFGLRGAVFADAGTLFGNKVSVGGGEYVNGEDASLRASVGVGLVWNSPFGALRV  754

Query  755  YYGIPLRHREGDKIQQFGFRIGNRM  779
             Y  P+   + DK+Q F F I N+
Sbjct  755  DYAFPVLKEDYDKVQHFKFGINNQF  779
```

b)

```
Identities:107/252(42%), Positives:158/252(62%), Gaps:3/252(1%)

Query  1    VPITESISTSFKYDLRFLQYGAISEKEKIPSIYTTLIEHGKFSSHSISQSIIYNTLDNPI  60
             PITE ++T+F+Y+ + ++Y        + + Y  L+E+  +   S+SQ++ YNTLD+
Sbjct  527  APITEDLATTFRYNYKQMKYDPSGSLSNLSAPYQNLVENSPWVKSSVSQTLTYNTLDDMT  586

Query  61   VPRKGMLISSSYDYAGFGGDSQYHRIGSRASYFYLLSDDSDIVGSLRFGYGCVIPSNKNL  120
            +PR+G+  S + + AG GGDSQ+++I  +A YF+LL+DD+DI+GSL    G V+  +KNL
Sbjct  587  LPREGIYASVTQEIAGLGGDSQFYKIYGKARYFHLLADDADIIGSLTGSAGYVVGFDKNL  646

Query  121  QLFDQFSVSSNYYLRGFAYKGIGPRV--DKKYAIGGKIYSSASAAVSFPMPLVPERAGLR  178
             +FDQF++ +N  +RGF  KGIGPR+       +GG  Y + SA  +FP+PLVP   GLR
Sbjct  647  NVFDQFTL-TNGDIRGFENKGIGPRIAGSNDDPLGGTTYFTVSAEATFPLPLVPRDFGLR  705

Query  179  GAFFVDSATLYANHVALGADKLEGNDSFWRVSTGVEIMWNSPLGMMGVYYGIPLRHREGD  238
            GA F D+ TL+ N V    D + G D+  R S GV +MW SP G + V Y IP+   + D
Sbjct  706  GALFADAGTLFGNDVDPLGDTVNGEDASLRASVGVGLMWASPFGTLRVDYAIPVAKEDFD  765

Query  239  KIQQFGFRIGNR  250
            K Q F F I N+
Sbjct  766  KTQHFKFGINNQ  777
```

Figure S5 – BLAST sequence comparison of CLas BamA (query) and *R. Grahamii* BamA (subject) proteins, for a) the full protein and b) extracellular and transmembrane region

Supplementary videos and additional information

Trajectory videos and simulation interaction reports for the molecular dynamics (MD) simulations have been uploaded to Zenodo and can be accessed using this link

https://zenodo.org/records/15858791
